# Lingonberry Leaves Modify Rumen Protozoa Population, Carbohydrate Digestion, and Morphology of Gastrointestinal Tract in Sheep: A Preliminary Study

**DOI:** 10.3390/molecules30153161

**Published:** 2025-07-29

**Authors:** Małgorzata P. Majewska, Renata Miltko, Grzegorz Bełżecki, Marcin Barszcz, Misza Kinsner, Barbara Kowalik

**Affiliations:** Department of Animal Nutrition, The Kielanowski Institute of Animal Physiology and Nutrition, Polish Academy of Sciences, Instytucka 3, 05-110 Jabłonna, Poland; r.miltko@ifzz.pl (R.M.); g.belzecki@ifzz.pl (G.B.); m.barszcz@ifzz.pl (M.B.); m.kinsner@ifzz.pl (M.K.); b.kowalik@ifzz.pl (B.K.)

**Keywords:** condensed tannins, protozoa, polysaccharidases, short-chain fatty acids, histology, sheep

## Abstract

Leaves, the main by-product of lingonberry harvesting, can be effectively used as a functional feed additive due to their health-promoting properties. This study evaluated the effects of lingonberry leaf (LL) supplementation on rumen fermentation, protozoal populations, and gastrointestinal morphology in sheep. Eight one-year-old Polish Mountain Sheep ewes (mean body weight: 33 kg) were allocated to a control (basal diet; forage-to-concentrate ratio 60:40) or an experimental group (basal diet + 9.30 g/kg DM dried LLs) in a completely randomised design (n = 4 per group) over 34 days. Both diets were formulated to be isoenergetic and isonitrogenous. LL additive significantly reduced *Isotrichidae* protozoal counts (*p* < 0.001) and ruminal pectinolytic activity (*p* = 0.043), without altering short-chain fatty acid (SCFA) or methane concentrations (*p* > 0.1). Histological analyses showed increased ruminal papilla width and surface area (*p* < 0.001) and decreased duodenal villus height and muscular layer thickness (*p* < 0.01). Inflammatory lesions (reddish foci) were identified in the liver in both groups. These findings demonstrate that LL supplementation affected specific protozoal population, fibrolytic activity, and gastrointestinal morphology. Further study on a larger number of animals is recommended to validate the effects and assess the safety and efficacy of LLs as a dietary additive in ruminant nutrition.

## 1. Introduction

In recent years, new dietary strategies with additives of natural origin have become popular in animal nutrition [1,2,3]. There is a growing emphasis on integrative approaches that simultaneously address the effects of dietary additives on digestive processes, animal health, and environment outcomes. Thus, scientists now prioritise identifying feed additives that would satisfy these multifactorial requirements and maintain safety standards.

**Figure 1:**
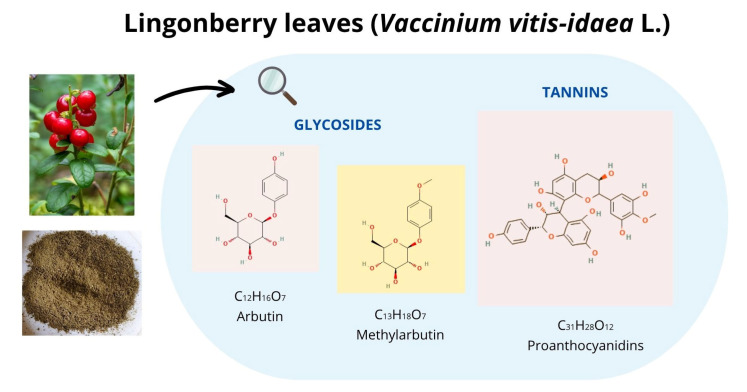
Lingonberry leaves (*Vaccinium vitis-idaea* L.) as a rich source of bioactive compounds (prepared by using https://canva.com). The chemical structure of the molecules was adopted from https://pubchem.ncbi.nlm.nih.gov (accessed on 30 May 2025).

Lingonberry (*Vaccinium vitis-idaea* L.), a member of the family *Ericaceae*, is a rich source of polyphenols with anti-inflammatory, antioxidant, and anticarcinogenic properties [4]. Leaves are the primary berry harvesting by-product and contain more polyphenols than the fruit [5], making them a promising candidate for use as a functional feed additive in animal nutrition. Proanthocyanidins are the predominant phenolic compounds in lingonberry leaves (Figure 1) [6,7,8]. These flavonoid-derived condensed tannins, with a C3-C6-C3 carbon skeleton lacking sugar residues, are resistant to acid, base, and microbial degradation [9]. Their biosynthesis occurs in chloroplast-derived tannosomes with storage in plant cells vacuoles [10]. In addition to tannins, lingonberry leaves also contain phenolic glycosides, such as arbutin and methylarbutin [11].

The rumen microbiota (bacteria, protozoa, and fungi) is involved in nutrient digestion [12], producing hydrolytic enzymes for plant carbohydrate digestion and short-chain fatty acids (SCFAs) as an energy source for ruminants [13]. Studies in ruminants have shown that tannin-rich plants influence ruminal digestion by modifying microbial populations and enzyme activity, which, in turn, affects nutrient availability throughout the gastrointestinal tract [14,15,16]. Tannins are recognised as inhibitors of microbial growth and development [14,17]. Their mode of action includes the formation of complexes with microbial cell walls, which interferes with nutrient transport and extracellular enzymes, thereby reducing their binding efficiency to feed particles [14]. While tannin–bacteria interactions are well documented [18,19,20], their effects on protozoa remain inconsistent, with both positive [21,22] and negative impacts [23,24] reported in the literature.

Tannins are classified as anti-nutritional factors that require careful dietary monitoring, as toxicity events has been documented in cases of high dietary tannin concentrations or excessive intake [25,26]. The literature data indicate that condensed tannins exert dose-dependent effects in ruminants. At high concentrations (>50 g/kg DM), tannins reduce feed intake, impair fibre digestion, and increase nitrogen excretion by forming insoluble complexes with proteins [27]. In contrast, low concentrations (5–10 g/kg DM) have been associated with increased post-ruminal protein availability and reduced methane production and bloat risk [27]. Tannin contents vary substantially between plants and depend on the species, growth stage, distribution area (altitude and latitude), environmental factors (light exposure, precipitation intensity, seasonal changes, and temperature), and storage conditions [10].

A key research challenge involves identifying doses that modulate physiological processes without inducing toxic effects. The effect of LL supplementation on ruminant health is unknown. There is also a lack of research examining the impact of an LL diet on the gastrointestinal morphology in ruminants. The present study on a small number of animals is an introduction to conduct wider multidirectional research on the effects of LL supplementation on sheep as a model for ruminants. The present study employed LL doses established in previous research [16,21].

This preliminary experiment tested the hypothesis that dietary supplementation with dried and milled lingonberry leaves (LLs) would affect protozoan population, carbohydrate fermentation, gas production in the rumen, and gastrointestinal morphology in sheep.

The objective was to determine the effects of 9.30 g/kg DM of dried LLs per day on protozoal counts, polysaccharidase activity, SCFA concentration, and associated methane production, as well as morphological characteristics of the rumen, duodenum, and liver.

## 2. Results

The dried LLs used in the study contained 167 mg of phenol equivalent/g dry weight (DW), 19.5 mg of catechin equivalent/g DW, and 4.52 mg of tannic acid equivalent/g DW, representing total phenols and condensed and hydrolysable tannins, respectively. Daily feed intake averaged 2.80% of live body weight, with no orts observed.

The experimental diet significantly reduced the number of *Isotrichidae* protozoa in the ruminal fluid, including *Isotricha* spp. and *Dasytricha* ssp. (*p* < 0.001; Table 1), and decreased pectinolytic activity (*p* = 0.043; Table 2).

No significant effects were observed on total SCFA concentration (*p* > 0.05); however, isoacid levels showed a decreasing trend (*p* = 0.069; Table 3). Methane concentration was not significantly affected by the treatment (*p* = 0.663).

Morphological measurements of the ruminal and duodenal mucosae showed that LL supplementation significantly increased ruminal papilla width and surface area (*p* < 0.001) while decreasing duodenal villus height and thickness of the muscular layer and increasing villus width (*p* < 0.001; Table 4).

**Figure 2:**
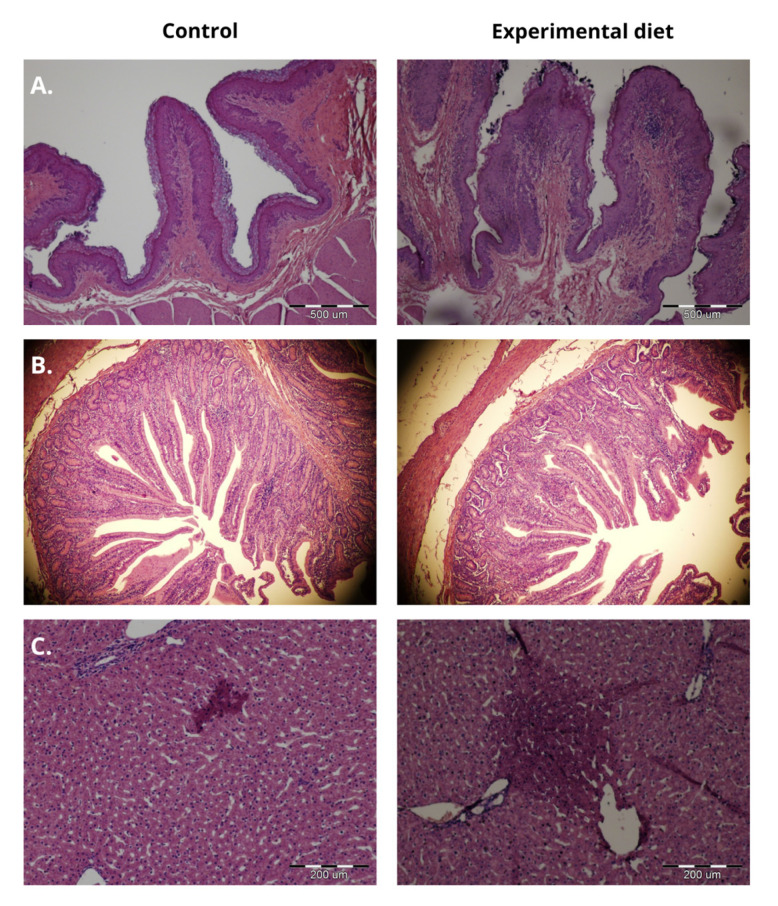
Effects of control diet and experimental diet with 10 g of LLs (*Vaccinium vitis-idaea* L.) addition on morphology of rumen, duodenum, and liver. (**A**) Rumen. Scale bar, 500 μm. (**B**) Duodenum. Scale bar, 500 μm. (**C**) Liver. Scale bar, 200 μm.

Inflammatory changes in the liver in the form of reddish lesions were observed in the control and experimental groups but were larger in LL-fed animals (Figure 2).

## 3. Discussion

Total phenolic content in plant material is typically determined using the Folin–Ciocalteu method, with gallic acid as the internal standard [5,29,30]. In the present study, the total phenol content was 167 mg phenol/g, which was comparable to the value reported by Stefănescu et al. (140 mg GAE/g) [5]. The concentration of condensed tannins in dried LLs, expressed as catechin equivalents, was higher than that of hydrolysable tannins. The spectrophotometric results obtained for catechin concentration are consistent with HPLC-DAD-ESI-MS data reported by Stefănescu et al. [5]. Reported variations in LL bioactive compound concentrations reflect differences in extraction methods, solvents (water, ethanol, acetone, and so on), temperature, duration, and analytical techniques [31].

In the present study, protozoa from the genus *Entodinium* represented the dominant population in the rumen. The dietary treatment significantly reduced protozoal counts from the family *Isotrichidae* (*Isotricha* spp. and *Dasytricha* ssp.), which are involved in the degradation of readily fermentable carbohydrates such as starch [13]. Similar results were obtained by Carulla et al. [23], where *Acacia mearnsii* extract (condensed tannins) selectively decreased the abundance of *Isotrichidae* in sheep. The proposed mechanism responsible for this finding involves tannin-starch complexation, limiting substrate availability for protozoal growth. The existing literature reports inconsistent effects of tannins on rumen protozoa, likely due to variations in tannin source, type, and dosage. Our previous study showed that supplementation with 2.80 g/kg DM of dried LLs reduced the abundance of *Diplodinium* spp. but increased the number of *Ophryoscolex* spp. in the sheep rumen [21]. Other studies involving plant extracts containing both condensed and hydrolysable tannins reported a general reduction in protozoal populations [32,33,34], potentially attributable to the higher polyphenol concentrations versus dried plant material. Several studies have also reported no significant impact of tannin-rich plants on protozoa in sheep [22,35]. The limited sample size and individual variability in this preliminary study likely explain the absence of a significant effect on protozoa other than *Isotrichidae*, particularly the predominant genus *Entodinium*.

Structural carbohydrates are building blocks of plant cell walls and an energy source for ruminants. The reduced pectinolytic activity observed in LL-fed sheep may result from the direct action of condensed tannins, which form complexes with pectinase and pectin itself. The binding capacity of tannins is influenced by their chemical structure (degree of polymerisation) and enzyme affinity [27,36]. Our prior research in sheep supplemented with hydrolysable tannins showed reduced amylolytic activity in the ruminal digesta, without affecting fibrolytic functions [16]. Here, no significant effects of dietary LL treatment were observed on other enzymatic activities. These findings align with those of Björck and Nyman [37] and Ozkose et al. [38], who observed decreased fibrolytic enzyme activity following tannic acid (a hydrolysable tannin) addition. In the present study, the number of protozoa specialising in fibre digestion (*Diplodinium* and *Ophryoscolex* genera) was not significantly affected, likely due to inter-animal variability. The present study did not assess LL impact on ruminal bacteria or fungi. However, existing research demonstrates that tannins can influence fibrolytic microbes: Cheng et al. [39] reported that protozoa digested only 30% of fibre, while Bae et al. [40] showed that condensed tannins impaired *Fibrobacter succinogenes* adhesion to feed particles, extracellular enzyme activity, and fibre digestion. Future studies should examine LL effects on broader microbial groups to comprehensively assess its impact on carbohydrate digestion.

Tannins form complexes with proteins (dietary, microbial, and endogenous), carbohydrates, and metal ions, potentially inhibiting nutrient digestion [41]. LL supplementation reduced the counts of *Isotrichidae* protozoa and pectinolytic activity, but these changes did not significantly affect total SCFA concentrations. Only a tendency towards decreased isoacid levels was observed. As branched-chain fatty acids are derived from ruminal amino acid deamination [42], their reduced concentration suggests lower protein degradation in the rumen following LL supplementation. Similar findings were reported by Salami et al. [34], who showed no significant fermentation changes with 4% tannin inclusion from various sources. Conversely, the studies of Cieślak et al. [32,41,42,43] documented increased propionic acid and decreased acetic acid concentrations after adding LL (condensed tannins) and oak bark (hydrolysable tannins) extracts. Additionally, Carrula et al. [23] recorded higher levels of butyric and valeric acids after using *Acacia mearnsii* extract (condensed tannins) in sheep diets. These discrepancies likely reflect differences between whole plant material and extracted tannin preparations.

Altered ruminal SCFA production affects methane generation. Previous studies have demonstrated methane reduction following plant extract supplementation [32,43,44]. Condensed tannins suppress methanogenesis through three mechanisms: (1) fibre complexation promoting propionate production (a methane antagonist), (2) direct inhibition of methanogen growth, and (3) reduced protozoal populations, which provide hydrogen to methanogens [45,46,47]. The dose of LLs applied in the present study was insufficient to reduce the methane concentration in the rumen. Similarly, the levels of acetic and propionic acids and the total number of protozoa were not significantly affected by LL supplementation. Since methane reduction is generally associated with limited nutrient digestibility, especially fibre, determining optimal doses that minimise production without compromising animal performance requires further investigation. Tedeschi et al. [46] emphasised that although methane-reducing properties of plant extracts are well documented, empirical evidence on their long-term action is still limited. In the present study, methane concentration was estimated based on the SCFA profile; thus, further in vivo studies involving respiration chambers are recommended.

The ruminal epithelium is responsible for nutrient absorption and transport, SCFA metabolism, and maintenance of rumen barrier integrity [48], and its development depends on nutrient intake, especially dietary carbohydrates and protein [49]. Increased ruminal size and density of papilla correlate with higher propionate and butyrate concentrations [49,50,51]. In the present study, LL addition to sheep diets increased the width of papilla and, thus, their surface area, which can improve SCFA absorption to the circulatory system. Redoy et al. [50] also showed an increased size of the papilla in sheep receiving plantain herb, garlic leaf, and their combination, attributing these changes to elevated propionic acid concentrations mediated by shifts in the microbial population. However, unlike the latter study, LL supplementation did not affect SCFA concentrations, likely because condensed tannins formed complexes with nutrients that limited direct interaction with epithelial tissues [52]. The near-neutral pH in the rumen favours the formation of such complexes. Keratinised, stratified squamous epithelium was observed in both dietary groups, although a thicker layer was reported in control sheep, potentially restricting nutrient transport [51]. This contrasts with findings in Boer goats, where high tannin intake from wattle extract increased keratinisation and impaired rumen function [53].

The small intestine plays a major role in nutrient digestion and absorption and is the largest immune organ in the body. Thus, its morphology is considered an important indicator of ruminant health [54]. In the current study, dietary LL inclusion negatively affected the morphology of the duodenum, reducing both villus height and muscular layer thickness. These changes may limit the absorptive surface of the villi and impair nutrient uptake from the intestinal lumen to the blood stream. Additionally, the thinner muscular layer observed in LL-fed sheep may reduce intestinal peristalsis and slow the passage of digesta through the intestine. One possible explanation for the observed alterations is the release of tannins from macromolecular complexes, leading to the accumulation of unbound tannins, which directly affect the duodenal epithelium. According to the literature, rapid pH changes in the gastrointestinal tract, including a decrease in the abomasum (pH < 3.5), followed by a rise in further sections of the small intestine (pH > 7) cause the dissociation of tannin–nutrient complexes [52]. Mbatha et al. [53] observed shortened height of villi, their erosion, and epithelial cell loss after increased doses of wattle extract in the diet, which induced lesions and impaired nutrient absorption. In those animals, increased mucus synthesis by goblet cells was noted as an adaptive mechanism to elevated levels of free condensed tannins in the gastrointestinal tract. Conversely, Zhao et al. [55] demonstrated that dietary inclusion of 0.1% tannic acid (a hydrolysable tannin) improved intestinal morphology by increasing villus height in the duodenum, jejunum, and ileum in Hu sheep.

Tannin toxicity depends on several factors, including their botanical origin, chemical structure, and dietary dose [26,56,57]. Condensed tannins with a complex structure remain undigested throughout the gastrointestinal tract [41], while compounds of low degree of polymerisation can be absorbed into the bloodstream. Low-molecular-weight tannin metabolites are considered toxic for animals [25]. Pérez et al. [26] documented mild hepatocellular lesions in cattle after oak leaf poisoning. In the current investigation, localised inflammatory changes, characterised by reddish discoloration, were observed in the livers of sheep fed the LL-supplemented diet, while occasional hepatocytes showed nuclear condensation and dissolution. Despite these inflammatory signs, the presence of mitotic figures indicated ongoing hepatic regeneration. Control animals displayed similar, though less pronounced, inflammatory changes (Figure 2). Based on the histological analyses, LL hepatotoxicity cannot be conclusively established. Liver inflammation in ruminants can lead to many negative health and production consequences, including metabolic disorders, reduced productivity, and impaired feed efficiency. Future studies should incorporate larger sample sizes and include hepatic biochemical markers to thoroughly assess the suitability of LLs in ruminant nutrition.

## 4. Material and Methods

Generative artificial intelligence (GenAI) has not been used in this paper.

### 4.1. Animal Diets

The study was performed on 8 one-year-old Polish Mountain Sheep ewes with an average body weight of 33 ± 1.1 kg (location: Jabłonna Country, Masovian Voivodeship, Poland, 52.378674° N, 20.908751° E), using a completely randomised design. The animals were randomly divided into 2 groups with 4 animals per group. All sheep were purchased from one breeder and were of the same breed, sex, age, and similar body weight to ensure group uniformity. Control sheep received a diet consisting of meadow hay, soybean meal, barley meal, and a mineral-vitamin premix (Table 5). The experimental group received the same base diet with the addition of 9.30 g/kg DM of dried and milled LLs (*Vaccinium vitis-idaea* L.) per day as a source of bioactive compounds, including condensed tannins. The plant material was purchased from the herbal supplier ‘KAWON-HURT’ Nowak sp. j. (Gostyń, Poland, license number IL 3333/LN). Diets were formulated according to IZ PIB-INRA recommendations for small ruminants [58] and were isonitrogenous and isoenergetic, containing approximately 15% crude protein and 2% crude fat. Animals were fed twice a day at 7.00 a.m. and 3.00 p.m. They were housed in a heated facility, equipped with gravitational ventilation (10 air exchanges per hour), natural daylight (window-to-floor area ratio ≥ 1:20), and artificial lighting. Temperature and humidity were monitored daily. The sheep were kept in individual pens with rubber mats, placed side by side to allow for visual and olfactory contact. Animals were able to move freely, lie, and stand up without restriction. The experimental trial lasted 34 days and consisted of three phases: adaptation period (12 days), dietary treatment (21 days), and sampling (1 day). During the adaptation phase, sheep in the experimental group were fed dried LLs at half the target dose, which was gradually increased to the full dose. All animals had ad libitum access to the fresh water and salt licks. Feed consumption was recorded daily. The animals were under constant veterinary care and their health status was monitored.

The samples of feed (forage, concentrate, and LLs) were taken for further chemical analysis, including the determination of dry matter (DM, 934.01); crude protein (954.01); starch (920.4); crude fibre (978.10); neutral detergent fibre (NDF, 2002.04); acid detergent fibre (ADF, 973.18); acid detergent lignin (ADL, 973.18), and crude ash (930.05), according to the methods of AOAC [59].

### 4.2. Slaughter Procedure and Sampling

Sheep were starved for 12 h but had free access to water. This procedure complies with both Polish [60,61] and EU [62] laws. The animals were slaughtered according to the standard commercial procedure [63]. Ruminal fluid and digesta were immediately collected from the dorsal and ventral sacs of the rumen and mixed precisely to obtain representative material for further analysis [64]. Rumen, duodenum, and liver samples were collected for further histological assessment.

### 4.3. Analyses of Bioactive Compounds in LLs

#### 4.3.1. Analysis of Total Phenols Concentration

**Figure 3:**
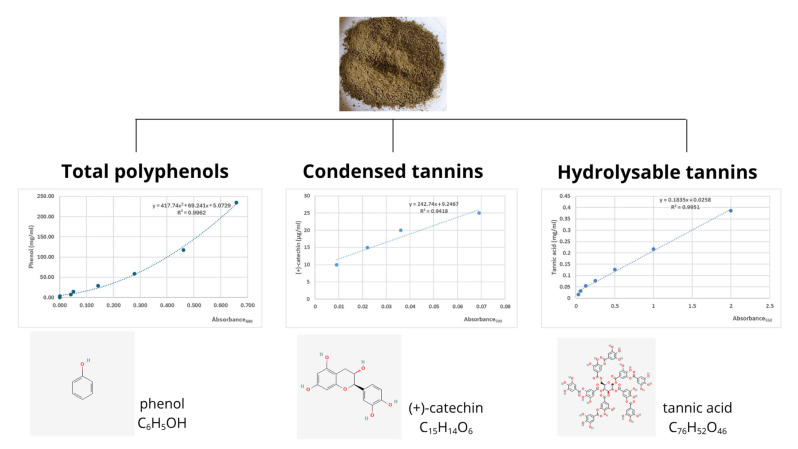
Bioactive compounds spectrophotometrically identified in dried milled lingonberry leaves (*Vaccinium vitis-idaea* L.). On this basis, the concentrations of total polyphenols and condensed and hydrolysable tannins in plant material were determined (prepared by using https://canva.com). The chemical structure of basic units was adopted from https://pubchem.ncbi.nlm.nih.gov (accessed on 20 June 2025).

A sample of dried and milled LLs (1.0 g) was mixed with 50 mL of double-distilled water for 1 h on a magnetic stirrer heated to 50 °C. The extract was then centrifuged at 3350× *g* for 10 min at room temperature, and 250 μL of the supernatant, diluted ten times in water, was taken for the deproteinisation and neutralisation. Total phenol concentration was measured spectrophotometrically at 690 nm using Folin–Ciocalteu’s reagent (Sigma-Aldrich, St. Louis, MO, USA) according to Barszcz et al. [65]. The concentration of phenolic compounds was calculated from a standard curve using serial dilutions of phenol (Sigma-Aldrich, St. Louis, MO, USA) and expressed as mg of phenol equivalent/g DW of LLs (Figure 3).

#### 4.3.2. Analysis of Condensed Tannins Concentration

A sample of dried and milled LLs (0.1 g) was mixed with 20 mL of dimethylformamide and 5 mL of sodium metabisulphite, cooked under the inverted chiller for 10 min and filtered. The 1 mL of extract obtained was collected into 2 glass test tubes and mixed with 9 mL of vanilin–H_2_SO_4_ solution (1st tube) and 9 mL of 70% H_2_SO_4_ solution (2nd tube) and incubated in a water bath at 20 °C for 15 min according to Kuhl and Ebmeier [66]. The condensed tannin concentration was determined spectrophotometrically at 500 nm and calculated according to the (+)-catechin (Sigma Aldrich, St. Louis, MO, USA) standard curve. The concentration of condensed tannins was expressed as mg of catechin equivalent/g DW of LLs (Figure 3).

#### 4.3.3. Analysis of Hydrolysable Tannins Concentration

A sample of dried and milled LLs (50 mg) was mixed with 10 mL of 70% aqueous acetone on a magnetic stirrer heated to 45 °C for 15 min. The extract obtained was centrifuged at 3350× *g* for 10 min at room temperature, diluted ten times in 70% aqueous acetone, saved, and stored in the refrigerator for further colorimetric analysis. Then, 5 mL of 2.5% aqueous solution of iodate potassium was incubated with 1 mL of plant extract or standard in a water bath at 25 °C for 15 min according to Willis and Allen [67]. After that, the hydrolysable tannin content was determined spectrophotometrically at 550 nm. Pure tannic acid served as a standard (Sigma Aldrich, St. Louis, MO, USA), and based on its serial dilutions, the concentration of hydrolysable tannins was calculated (mg of tannic acid equivalent/g DW of LLs) (Figure 3).

### 4.4. Determination of Protozoa Number

The samples of ruminal fluid were filtered through 2 layers of surgical gauze to remove large particles of food. Then, 5 mL of fluid was fixed with 10 mL of 4% aqueous formaldehyde solution [21]. The obtained samples were stored at 4 °C in the tightly closed containers until analysis. The protozoa number was identified based on morphological criteria as described previously by Dehority [68] and Miltko et al. [69], including the number and size of ciliary zones, the number and location of contractile vacuoles, the number of spines/lobes, as well as overall size and shape of the cell. To determine the number of protozoa, each sample was counted under a light microscope in 2 replications (Table S1). For this purpose, the samples analysed were diluted, and 0.1 mL was poured on the microscopic glass slide and spread on the slide with a needle.

### 4.5. Enzymatic Analysis

The polysaccharidases were extracted from the ruminal digesta in the presence of 1% phosphate buffer, carbon tetrachloride, and lysozyme according to the method briefly described by Miltko et al. [13]. Carboxymethylcellulose, beech wood xylan, potato starch, pectin from citrus, and inulin purchased from Sigma-Aldrich Co., (St. Louis, MO, USA) served as substrates for determining cellulolytic, xylanolytic, amylolytic, pectinolytic, and inulinolytic activities in the ruminal digesta, respectively. The amount of reducing sugars was measured on the spectrophotometer at 560 nm, and on this basis, the hydrolytic activity of polysaccharidases was determined (Table S2).

### 4.6. Determination of Short-Chain Fatty Acid Concentration

The SCFA concentration was determined by using a gas chromatograph (Shimadzu GC-2010, Tokyo, Japan) equipped with a capillary column (30 m length, 0.25 mm i.d. × 0.25 μm film thickness) and flame ionisation detector (FID) according to Miltko et al. [70]. The temperature of the injector and FID were maintained at 250 and 280 °C, respectively. Helium served as the carrier gas, with an initial pressure of 37.3 kPa and constant flow rate (0.87 mL/min). The flow rates of H_2_ and air were maintained at 40 and 400 mL/min, respectively. Briefly, the samples of filtered ruminal fluid were treated with 85% formic acid, and after 30 min, they were centrifuged at 11,000× *g* for 25 min in 4 °C. The supernatant obtained was placed in the vials and stored in the refrigerator until analysis. Before analysis, 1 mL of ruminal fluid was placed into the 2 mL Eppendorf tubes and centrifuged at 6000× *g* for 15 min to remove all residuals. Then, 0.5 mL of the supernatant obtained was taken into the vials, and 75 μL of 4-methylvaleric acid was added as an internal standard (277827, Sigma-Aldrich Co., St. Louis, MO, USA). Such prepared samples were analysed on a gas chromatograph. The SCFA concentration was determined using 1 µL of sample at a split ratio of 10:1 according to the following column temperature programme: 80 °C for 1 min, increasing by 15 °C/min to 220 °C, and maintained for 4 min.

**Figure 4:**
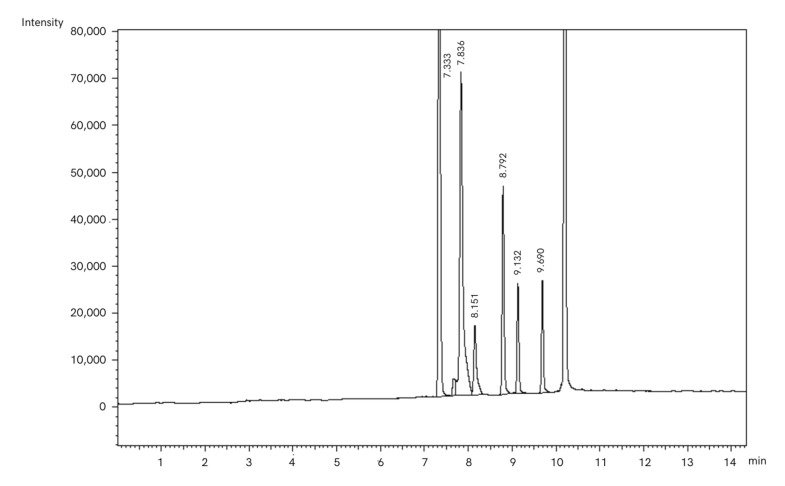
The example of selected chromatogram presenting peaks of SCFAs: acetic acid (RT, retention time—7.333 min), propionic acid (RT—7.836 min), iso-butyric acid (RT—8.151 min), butyric acid (RT—8.792 min), iso-valeric acid (RT—9.132 min), and valeric acid (RT—9.690 min).

All peaks of FA appearing on chromatograms (Figure 4) were identified according to the standards provided: acetic acid (695092), propionic acid (402907), iso-butyric acid (I1754), butyric acid (B103500), iso-valeric acid (129542), and valeric acid (240370), purchased from Sigma-Aldrich Co., (St. Louis, MO, USA). Finally, the peaks were integrated by using GC software 112 (LabSolutions, Shimadzu, Tokyo, Japan; Table S3).

### 4.7. Methane Production

Based on the concentrations of the selected SCFAs, the methane concentration in the rumen was estimated (Table S4) according to the following equation:

Methane=0.45×C2 −0.275×C3+0.40×C4

where *C*2, *C*3, and *C*4 represent the concentrations of acetic, propionic, and butyric acids, respectively [28].

### 4.8. Histological Analyses

Tissue samples from the rumen, duodenum, and liver were fixed in 10% buffered formalin, dehydrated, and embedded in paraffin. For histological analyses, each sample was sliced into 5 μm sections on two slides on a Microm 350 rotary microtome (Thermo Fisher Scientific, Walldorf, Germany) and stained with haematoxylin and eosin. Obtained slides were analysed on an Olympus BX51 light microscope (Olympus Corp., Tokyo, Japan) at 10× and 40× magnification, with the use of Cell^D^ Imaging Software (Olympus Soft Imaging Solutions, Munster, Germany). At least 10 measurements of each parameter were described. According to Odongo et al. [71], in the ruminal mucosa preparations, papilla height, papilla width, and its surface area (calculated as papilla height × papilla width) were determined (Table S5). Regarding duodenal preparations, villus height and width, crypt depth, and thickness of the muscular layer were examined (Table S6). By contrast, liver tissue samples have been analysed for any histopathological changes, such as inflammation, necrosis, or degeneration, in order to assess whether LL has a toxic effect on sheep.

### 4.9. Statistical Analyses

The results are presented as means with pooled standard deviations for the two groups (SDs). Data normality was assessed using the Shapiro–Wilk test. Data with normal distribution were analysed using the independent *t* test, while non-normally distributed data were evaluated using Mann–Whitney U test for two independent groups. This model included the effect of dietary treatment (LL supplementation). Statistical significance was set at *p* < 0.05. Trends were also reported and discussed for values where 0.05 < *p* < 0.10 (StatSoft^®^, Kraków, Poland). For all parameters, Cohen’s d was calculated to measure of size effect of dietary treatment.

Sample size adequacy was determined by power analysis using Statgraphics Centurion XVI ver. 16.1.03 (StatPoint Technologies, Inc., Warrenton, VA, USA) based on previous experiments in sheep (protozoal count, enzymatic activities, SCFA concentrations, and rumen histology). The analysis was performed at 95% power with α = 0.05, including the hypothesised mean, standard deviations, and minimum detectable differences. For the total number of protozoa, the minimum detectable difference was set at 20, with a standard deviation of 5. For cellulolytic activity, these were 3.0 and 2.0; for pectinolytic activity and total SCFA concentration, 3.0 and 1.0; for butyrate concentration, 0.3 and 0.2; for rumen papilla height, 0.5 and 0.2; and for rumen papilla width, 0.08 and 0.02, respectively. An average sample size was subsequently calculated based on these parameters.

## 5. Conclusions

This preliminary in vivo study on sheep evaluated the potential application and safety of lingonberry leaves (LLs) in sheep nutrition. Dietary inclusion of 9.30 g/kg DM of LLs significantly reduced *Isotrichidae* protozoal populations and pectinolytic activity without affecting short-chain fatty acid (SCFA) concentrations or estimated methane production in the rumen. Furthermore, LL addition significantly altered ruminal and duodenal morphologies, indicating potential impacts on nutrient absorption efficiency. Histopathological examination revealed hepatic inflammatory changes in both control and experimental groups, precluding definitive conclusions regarding LL-specific toxicity.

The limitations of the current experimental design require conducting further research involving a larger number of animals to confirm these findings and comprehensively assess the efficacy and safety of LLs in sheep nutrition. Future research should also include in vivo methane measurements using respiration chambers and expanded biochemical blood analyses to evaluate potential hepatic effects.

## Figures and Tables

**Table 1:** Protozoa number in the ruminal fluid of sheep (×10^4^/mL).

Item	Control	Experimental Diet	SD	*p*-Value	Cohen’s *d*
Total protozoa	163.7	140.0	24.86	0.389	0.953
*Entodinium*	143.6	127.9	24.74	0.554	0.613
*Diplodinium*	4.9	4.9	0.40	0.860	−0.193
*Ophryoscolex*	1.9	1.3	0.40	0.167	1.630
*Isotricha*	2.4 ^A^	1.3 ^B^	0.16	<0.001	7.038
*Dasytricha*	10.9 ^A^	4.6 ^B^	0.62	<0.001	8.214

SD, pooled standard deviation for the two groups; ^AB^ means with different superscripts in a row differ significantly at *p* ≤ 0.05.

**Table 2:** Polysaccharidase activities of the ruminal digesta of sheep (μM carbohydrate released ‧ g^−^^1^ DM ‧ min^−^^1^).

Enzyme Activity	Control	Experimental Diet	SD	*p*-Value	Cohen’s *d*
Cellulolytic ^1^	41.4	37.4	4.08	0.210	0.994
Xylanolytic ^2^	60.0	56.8	4.95	1.000	0.633
Amylolytic ^1^	47.7	40.7	3.38	0.167	1.249
Pectinolytic ^3^	6.5 ^A^	4.5 ^B^	1.14	0.043	1.814
Inulinolytic ^4^	7.7	7.6	0.75	0.850	0.140

SD, pooled standard deviation for the two groups; ^1^ μΜ glucose released ‧ g^−1^ DM ‧ min^−1^; ^2^ μM xylose released ‧ g^−1^ DM ‧ min^−1^; ^3^ μM glucuronic acid released ‧ g^−1^ DM ‧ min^−1^; ^4^ μM fructose released ‧ g^−1^ DM ‧ min^−1^; ^AB^ means with different superscripts in a row differ significantly at *p* ≤ 0.05.

**Table 3:** SCFA concentration and methane production in the ruminal fluid of sheep (mM/100 mL).

Item	Control	Experimental Diet	SD	*p*-Value	Cohen’s *d*
Total SCFA	6.3	7.8	1.91	0.369	−0.825
Acetate	4.4	5.7	1.50	0.361	−0.839
Propionate	0.8	0.9	0.17	0.437	−0.879
Butyrate	0.6	0.9	0.27	0.176	−1.307
Valerate	0.06	0.05	0.01	0.576	0.707
Isoacids ^1^	0.3	0.2	0.03	0.069	1.820
Methane ^2^	2.0	2.7	0.63	0.663	−1.045

SD, pooled standard deviation for the two groups; SCFA, short-chain fatty acid; ^1^ sum of iso-butyric and iso-valeric acids; ^2^ calculated according to Moss et al. [28].

**Table 4:** Morphological indices of the ruminal and duodenal mucosae of sheep.

Item	Control	Experimental Diet	SD	*p*-Value	Cohen’s *d*
Rumen					
Papilla height, mm	1.5	1.6	0.60	0.108	−0.045
Papilla width, mm	0.3 ^B^	0.4 ^A^	0.08	<0.001	−1.014
Papilla surface area, mm^2^ *	0.5 ^B^	0.6 ^A^	0.23	<0.001	−0.644
Duodenum					
Villus height, μm	1078.2 ^A^	879.5 ^B^	154.71	<0.001	1.284
Villus width, μm	116.1 ^B^	124.5 ^A^	13.89	<0.008	−0.609
Crypt depth, μm	168.1	167.8	31.11	0.967	0.009
Thickness of the muscular layer, μm	259.4 ^A^	189.7 ^B^	50.57	<0.001	1.379

SD, pooled standard deviation for the two groups. * Calculated as papilla height × papilla width. ^AB^ Means with different superscripts in a row differ significantly at *p* ≤ 0.05.

**Table 5:** Composition of sheep diets.

Item	Control	Experimental Diet
Components (g/kg DM)		
Meadow hay	548.0	548.0
Barley meal	260.6	260.6
Soybean meal	90.3	90.3
Polfamix O-K ^1^	19.0	19.0
Lingonberry leaves (LLs)	-	9.3
LL composition (mg/g DW)		
Total phenols ^2^	-	167
Condensed tannins ^3^	-	19.5
Hydrolysable tannins ^4^	-	4.5
Chemical composition (g/kg DM)		
Dry matter	899.9	900.2
Organic matter	846.8	847.3
Crude protein ^5^	136.9	136.2
Crude fat	18.0	18.6
Starch	222.1	220.0
Crude fibre	182.7	182.6
NDF	460.2	459.3
ADF	233.5	234.1
ADL	35.8	37.6
Crude ash	34.6	34.6
LL chemical composition (g/kg DM)		
Dry matter	-	9.0
Organic matter	-	8.8
Crude protein ^5^	-	0.6
Crude fat	-	0.6
Crude fibre	-	1.7
NDF	-	3.6
ADF	-	2.8
ADL	-	1.6
Crude ash	-	0.3
Nutrient intake (g/d)		
Dry matter	917.9	927.2
Organic matter	863.7	872.7
Crude protein	139.6	140.3
Crude fat	18.3	19.1
Starch	226.6	226.6
Crude fibre	186.3	188.0
NDF	469.4	473.1
ADF	238.2	241.1
ADL	36.5	38.7
Crude ash	35.3	35.6

DM, dry matter; NDF, neutral detergent fibre; ADF, acid detergent fibre; ADL, acid detergent lignin; ^1^ Polfamix O-K (Trouw Nutrition, Grodzisk Mazowiecki, Poland) in kg: g: Ca 240, Na 60, P 120, Mg 65, Zn 2.5, Mn 3.0, vit. E 1.5, Se 0.003, and Co 0.015; IU: vit. A 300,000 and vit. D3 30,000; ^2^ mg of phenol equivalent/g DW of LLs; ^3^ mg of (+)-catechin equivalent/g DW of LLs; ^4^ mg of tannic acid equivalent/g DW of LLs; ^5^ expressed as N × 6.25.

## Data Availability

The original contributions presented in this study are included in the article. Further inquiries can be directed to the corresponding author.

## Supplementary Materials

The following supporting information can be downloaded at: https://www.mdpi.com/article/10.3390/molecules30153161/s1, Table S1: Protozoa number (×10^4^), raw data; Table S2: Polysaccharidase activity, raw data; Table S3: Short-chain fatty acid concentration, raw data; Table S4: Estimated methane concentration, raw data; Table S5: Ruminal morphology, raw data; Table S6: Duodenal morphology, raw data.

## Abbreviations

The following abbreviations are used in this manuscript:

ADF	Acid detergent fibre
ADL	Acid detergent lignin
DM	Dry matter
DW	Dry weight
LLs	Lingonberry leaves
NDF	Neutral detergent fibre
RT	Retention time
SCFA	Short-chain fatty acid
SEM	Standard error of mean
