# Supplementary material for: Lingonberry Leaves Modify Rumen Protozoa Population, Carbohydrate Digestion, and Morphology of Gastrointestinal Tract in Sheep: A Preliminary Study
Source: Molecules. 2025 Jul 29;30(15):3161. doi: 10.3390/molecules30153161 (PMC12348859; doi:10.3390/molecules30153161)
Supplement: Supplementary file 1 [file molecules-30-03161-s001.zip › molecules-3730304-supplementary.pdf]

Table S1. Protozoa number (× 10^4), raw data.

Sheep number	Total protozoa	*Entodinium*	*Diplodinium*	*Ophyroscolex*	*Isotricha*	*Dasytricha*
1	150.4	131.56	4.88	1.16	2.48	10.32
	149.0	131.36	4.24	1.00	2.60	9.80
	**149.7**	**131.46**	**4.56**	**1.08**	**2.54**	**10.06**
2	140.3	131.40	5.10	1.68	1.08	3.92
	133.3	124.44	4.70	1.4	1.12	3.72
	**136.8**	**127.92**	**4.90**	**1.54**	**1.10**	**3.82**
3	173.2	151.0	4.73	1.82	2.0	10.96
	195.0	171.52	4.97	1.98	2.40	12.08
	**184.1**	**161.26**	**4.85**	**1.90**	**2.20**	**11.52**
4	201.8	189.44	5.48	1.16	1.40	4.32
	161.8	149.52	5.56	0.96	1.24	4.52
	**181.8**	**169.48**	**5.52**	**1.06**	**1.32**	**4.42**
5	133.2	112.12	4.40	2.84	2.56	11.28
	128.0	107.88	4.48	2.24	1.92	11.48
	**130.6**	**110.0**	**4.44**	**2.54**	**2.24**	**11.38**
6	119.6	106.76	4.64	1.16	1.32	5.72
	128.4	116.56	4.36	1.12	0.88	5.48
	**124.0**	**111.66**	**4.50**	**1.14**	**1.10**	**5.60**
7	190.6	169.08	6.0	2.28	2.48	10.76
	190.2	170.24	5.08	1.92	2.48	10.48
	**190.4**	**169.66**	**5.54**	**2.10**	**2.48**	**10.62**
8	118.0	102.8	4.72	1.68	1.40	4.40
	116.8	102.56	4.84	1.12	1.60	4.76
	**117.4**	**102.68**	**4.78**	**1.40**	**1.50**	**4.58**

Table S2. Polysaccharidase activity, raw data.

Sheep number	Cellulolytic	Xylanolytic	Amylolytic	Pectinolytic	Inulinolytic
1	35.3358	58.4468	45.2288	5.87044	7.1517
	35.4039	61.5636	44.4796	4.64950	7.5101
	**35.3698**	**60.0052**	**44.8542**	**5.2560**	**7.3309**
2	40.1564	59.8152	46.7124	5.3157	6.9468
	39.6158	58.7180	43.8848	5.6783	6.9329
	**39.8861**	**59.2666**	**45.2986**	**5.4970**	**6.9398**
3	42.8353	54.0239	46.2348	6.2510	7.3373
	42.0301	53.8916	49.2340	5.0216	7.0438
	**42.4327**	**53.9578**	**47.7344**	**5.6363**	**7.1905**
4	35.4777	54.3914	40.6196	4.3523	8.4388
	36.6563	54.3071	39.6611	4.5908	8.6985
	**36.0670**	**54.3493**	**40.1404**	**4.4715**	**8.5687**
5	48.1262	68.2779	54.2010	8.9725	7.5521
	47.5710	69.4131	52.9797	7.2511	8.1086
	**47.8486**	**68.8455**	**53.5904**	**8.1118**	**7.8304**
6	34.8555	54.8983	42.5340	3.2194	6.9175
	34.2573	53.7818	44.4772	3.3812	6.7641
	**34.5564**	**54.3400**	**43.5056**	**3.3003**	**6.8408**
7	39.7909	57.1594	44.5109	7.9497	8.0538
	40.8383	57.3320	45.0014	6.3438	9.0638
	**40.3146**	**57.2457**	**44.7562**	**7.1467**	**8.5588**
8	39.2651	59.8733	45.9418	4.7640	8.2779
	39.2491	58.8513	44.2487	4.5163	8.0057
	**39.2571**	**59.3623**	**45.0953**	**4.6401**	**8.1418**

Table S3. Short-chain fatty acid concentration, raw data.

Sheep number	Total	Acetic acid	Propionic acid	Butyric acid	Valeric acid	Isoacids
1	7.124	5.231	0.991	0.572	0.058	0.272
	7.129	5.309	0.945	0.549	0.062	0.264
	**7.127**	**5.270**	**0.968**	**0.561**	**0.060**	**0.268**
2	10.167	7.735	1.062	1.109	0.049	0.212
	10.165	7.685	1.083	1.129	0.050	0.218
	**10.166**	**7.710**	**1.073**	**1.119**	**0.050**	**0.215**
3	6.308	4.441	0.767	0.735	0.067	0.298
	6.117	4.308	0.750	0.713	0.062	0.284
	**6.213**	**4.375**	**0.759**	**0.724**	**0.065**	**0.291**
4	9.470	6.905	0.953	1.266	0.063	0.283
	9.922	7.153	1.022	1.376	0.072	0.299
	**9.696**	**7.029**	**0.988**	**1.321**	**0.068**	**0.291**
5	4.907	3.596	0.569	0.433	0.041	0.268
	4.849	3.535	0.554	0.431	0.038	0.291
	**4.878**	**3.566**	**0.562**	**0.432**	**0.040**	**0.280**
6	5.665	4.180	0.655	0.585	0.042	0.203
	5.751	4.214	0.679	0.604	0.040	0.214
	**5.708**	**4.197**	**0.667**	**0.595**	**0.041**	**0.208**
7	6.152	4.461	0.736	0.571	0.066	0.318
	6.090	4.379	0.724	0.589	0.072	0.326
	**6.121**	**4.420**	**0.730**	**0.580**	**0.069**	**0.322**
8	5.511	3.722	0.906	0.659	0.036	0.188
	5.455	3.721	0.874	0.639	0.033	0.188
	**5.483**	**3.722**	**0.890**	**0.649**	**0.035**	**0.188**

Table S4. Estimated methane concentration, raw data.

Sheep number	Methane
1	2.310
	2.349
	**2.330**
2	3.630
	3.612
	**3.621**
3	2.081
	2.018
	**2.049**
4	3.344
	3.481
	**3.413**
5	1.635
	1.610
	**1.623**
6	1.934
	1.951
	**1.943**
7	2.033
	2.007
	**2.020**
8	1.688
	1.689
	**1.689**

Table S5. Ruminal morphology, raw data.

Sheep number	Papilla height	Papilla width	Papilla surface area
1	1.1369	0.3178	0.3613
	1.2065	0.2452	0.2958
	0.9523	0.3896	0.3710
	1.1964	0.3362	0.4022
	0.8631	0.2298	0.1983
	0.7110	0.2354	0.1674
	1.1454	0.2597	0.2975
	1.1640	0.3429	0.3991
	1.1277	0.3170	0.3575
	0.9682	0.3907	0.3782
	1.1896	0.3111	0.3700
	0.8566	0.2237	0.1916
	0.8644	0.4305	0.3721
	0.8249	0.4557	0.3759
	0.7119	0.2382	0.1696
	1.1230	0.3077	0.3455
	0.9803	0.2545	0.2495
	1.1325	0.3144	0.3561
	1.2163	0.2687	0.3268
	0.9788	0.3748	0.3669
	1.1741	0.3243	0.3808
	**1.0249**	**0.3128**	**0.3206**
2	1.4362	0.4001	0.5746
	1.2256	0.6194	0.7591
	1.5562	0.5759	0.8962
	2.3985	0.7080	1.6982
	1.4329	0.3875	0.5552
	2.2160	0.4931	1.0927
	1.5881	0.5474	0.8693
	1.3941	0.4113	0.5734
	1.4819	0.5729	0.8490
	1.1451	0.5824	0.6669
	**1.5875**	**0.5298**	**0.8535**
3	1.1883	0.2737	0.3252
	1.1817	0.3194	0.3774
	0.9965	0.3111	0.3100
	1.2006	0.3533	0.4242
	1.0400	0.3487	0.3627
	1.2686	0.3513	0.4456
	0.9787	0.3320	0.3250
	1.0177	0.3356	0.3415
	1.3911	0.3304	0.4595
	1.1450	0.2782	0.3186
	1.1871	0.2933	0.3482
	1.0490	0.2933	0.3077
	1.2421	0.3621	0.4498
	1.0291	0.3355	0.3453

	0.9824	0.3537	0.3475
	1.2558	0.3479	0.4369
	1.0049	0.3454	0.3471
	1.0248	0.3498	0.3585
	1.0394	0.3629	0.3772
	1.3445	0.3435	0.4618
	1.1586	0.2832	0.3282
	1.0427	0.2950	0.3076
	1.1790	0.3471	0.4092
	1.2581	0.3797	0.4777
	0.9782	0.3392	0.3318
	**1.1274**	**0.3306**	**0.3730**
4	1.3678	0.4084	0.5585
	1.3270	0.3766	0.4998
	0.9607	0.3921	0.3767
	0.9842	0.3818	0.3758
	1.6938	0.4157	0.7041
	1.3929	0.3066	0.4271
	1.9938	0.3277	0.6534
	1.4390	0.4113	0.5919
	1.2062	0.4170	0.5029
	1.0026	0.3590	0.3599
	1.7175	0.4137	0.7106
	1.4011	0.4118	0.5769
	1.4385	0.4223	0.6075
	**1.3789**	**0.3880**	**0.5342**
5	2.0161	0.2853	0.5752
	2.25242	0.2081	0.4687
	1.87054	0.46166	0.8636
	2.38845	0.3471	0.8290
	1.29392	0.3103	0.4015
	2.85726	0.33052	0.9444
	1.52157	0.3303	0.5026
	2.29363	0.34569	0.7929
	1.87343	0.47112	0.8826
	2.01939	0.30804	0.6221
	2.30764	0.22068	0.5092
	1.81667	0.41831	0.7599
	2.26764	0.36923	0.8373
	1.32921	0.32023	0.4257
	1.32565	0.32412	0.4297
	1.8608	0.44257	0.8235
	1.98241	0.2876	0.5701
	**1.9575**	**0.3401**	**0.6611**
6	1.5261	0.3100	0.4730
	2.6874	0.3048	0.8191
	1.3510	0.2738	0.3699
	1.3469	0.4183	0.5634
	2.8960	0.2193	0.6351

	2.3816	0.3716	0.8850
	2.2187	0.3976	0.8821
	2.2466	0.3575	0.8030
	1.4991	0.2950	0.4423
	2.6583	0.3241	0.8615
	**2.0812**	**0.3272**	**0.6735**
7	1.9418	0.4728	0.9181
	2.2514	0.3361	0.7568
	3.7701	0.1938	0.7305
	3.5465	0.2227	0.7899
	2.6658	0.2618	0.6979
	1.4347	0.3378	0.4846
	1.9444	0.4683	0.9105
	2.8275	0.1893	0.5353
	2.7103	0.2492	0.6754
	1.3442	0.3123	0.4197
	2.3619	0.2997	0.7079
	1.3279	0.3186	0.4230
	2.7260	0.2406	0.6558
	2.3710	0.3388	0.8033
	1.2875	0.3235	0.4165
	2.4124	0.2814	0.6789
	2.7064	0.3100	0.8389
	2.1920	0.2253	0.4939
	**2.3234**	**0.2990**	**0.6632**
8	1.4457	0.3759	0.5435
	1.1703	0.4597	0.5379
	1.4465	0.3646	0.5274
	1.0119	0.4147	0.4196
	0.7856	0.2490	0.1956
	0.8273	0.4318	0.3572
	1.4279	0.3616	0.5164
	**1.1593**	**0.3796**	**0.4425**

Table S6. Duodenal morphology, raw data.

Sheep number	Villus height	Villus width	Crypt depth	Thickness of the muscular layer
1	1298.92	87.32	178.84	201.19
	1286.28	85.33	205.33	194.74
	1292.03	106.04	191.78	179.14
	1280.74	145.85	152.80	341.52
	1257.09	99.57	199.08	221.38
	1367.22	106.46	190.30	235.75
	1336.90	110.43	181.89	336.40
	1213.64	109.21	143.77	319.07
	1260.67	114.58	236.78	279.76
	1407.21	113.29	154.81	164.03
	**1300.07**	**107.81**	**183.54**	**247.30**
2	831.19	125.07	213.36	195.49
	927.27	120.30	141.02	184.51
	1019.03	102.75	146.74	201.8
	1010.50	146.54	180.78	208.74
	880.25	112.74	166.33	232.34
	918.75	122.91	175.18	146.51
	704.62	121.59	255.71	209.23
	862.53	117.49	199.04	167.21
	768.20	109.66	220.19	176.6
	862.32	122.34	200.33	191.26
	**878.47**	**120.14**	**189.87**	**191.37**
3	1015.95	125.45	197.20	214.37
	1047.26	117.18	128.38	224.37
	1133.65	121.89	207.99	302.77
	1147.11	107.46	153.14	312.53
	1181.23	125.79	148.79	229.16
	856.52	135.09	168.54	245.87
	944.61	130.00	159.33	237.81
	970.25	124.66	166.12	330.84
	1165.94	103.43	120.71	252.11
	1049.02	99.25	170.38	211.97
	**1051.15**	**119.02**	**162.06**	**256.18**
4	752.95	131.45	158.02	183.19
	884.13	117.94	156.65	191.88
	935.89	151.18	203.10	154.62
	812.36	123.07	157.79	139.50
	872.28	110.75	193.47	140.42
	863.69	112.96	139.24	145.50
	893.71	103.29	177.33	146.72
	912.59	107.82	204.01	192.05
	792.05	103.32	117.74	196.84
	823.18	138.82	178.53	160.31
	**854.28**	**120.06**	**168.59**	**165.10**

5	988.06	117.61	163.75	292.22
	981.72	121.25	129.59	343.83
	981.22	119.57	137.86	333.76
	888.83	134.58	166.49	273.36
	737.96	117.48	190.82	271.12
	753.95	130.96	165.09	269.22
	745.43	115.68	238.00	243.93
	703.06	106.23	212.03	270.05
	735.92	127.49	182.77	374.13
	855.72	109.36	181.53	306.35
	**837.19**	**120.02**	**176.79**	**297.80**
6	898.49	123.37	166.88	171.25
	856.9	128.34	167.72	177.61
	824.66	114.51	148.91	174.56
	565.13	145.51	194.12	163.65
	746.54	147.26	150.72	150.57
	759.98	139.72	186.49	139.54
	760.20	141.49	145.04	145.70
	808.86	123.20	199.95	151.59
	788.64	102.96	168.35	160.66
	978.31	131.31	119.25	179.09
	**798.77**	**129.77**	**164.74**	**161.42**
7	1170.06	123.78	145.01	166.66
	1089.63	100.81	146.48	189.22
	1127.11	110.70	138.58	206.48
	1082.55	107.57	159.08	171.83
	1042.24	100.55	222.34	164.32
	1076.40	110.66	165.08	285.96
	1149.77	121.82	143.59	306.92
	1125.42	132.62	107.75	267.69
	1179.37	125.87	123.66	367.99
	1200.03	139.59	149.38	237.61
	**1124.26**	**117.40**	**150.10**	**236.47**
8	853.27	140.66	131.13	198.49
	946.07	129.64	132.19	271.40
	879.68	111.08	154.65	257.93
	938.00	139.85	198.80	267.19
	1037.63	103.40	150.05	286.39
	1083.28	128.64	143.20	217.25
	1135.66	148.52	139.93	227.09
	1086.01	131.83	168.44	251.53
	968.54	114.94	111.71	250.77
	937.49	132.26	151.08	181.55
	**986.56**	**128.08**	**148.12**	**240.96**
